# Loneliness trajectories surrounding mobility loss: the role of disability duration

**DOI:** 10.1093/geronb/gbag155

**Published:** 2026-08-05

**Authors:** Erika Augustsson, Stefan Fors, Lena Dahlberg, Carin Lennartsson, Neda Agahi

**Affiliations:** Aging Research Center, Karolinska Institutet and Stockholm University, Stockholm, Sweden; Aging Research Center, Karolinska Institutet and Stockholm University, Stockholm, Sweden; Centre for Epidemiology and Community Medicine, Region Stockholm, Sweden; Aging Research Center, Karolinska Institutet and Stockholm University, Stockholm, Sweden; School of Health and Welfare, Dalarna University, Falun, Sweden; Aging Research Center, Karolinska Institutet and Stockholm University, Stockholm, Sweden; Swedish Institute for Social Research, Stockholm University, Stockholm, Sweden; Aging Research Center, Karolinska Institutet and Stockholm University, Stockholm, Sweden; (Social Sciences Section)

**Keywords:** Longitudinal trajectories, Disability duration, Mobility limitations

## Abstract

**Objectives:**

Mobility limitations in later life can disrupt social engagement and increase loneliness, but trajectories may differ depending on whether limitations remain or individuals recover. This study examines loneliness trajectories around the onset of mobility limitations, focusing on differences by limitation duration.

**Methods:**

Data from the Survey of Health, Ageing and Retirement in Europe were used to analyze 4,109 adults aged 65+ who experienced the onset of mobility limitations, of whom 2,018 recovered within 2 years. A reference group of 8,197 individuals had no mobility limitations. Loneliness was measured up to 2 years before and 4 years after the onset. Linear spline mixed-effects models estimated trajectories by onset and limitation duration.

**Results:**

Loneliness increased following the onset of mobility limitations. Individuals with persistent limitations showed continued increases, while those who recovered stabilized, though not returning to pre-onset levels.

**Discussion:**

These findings suggest that early interventions to mitigate mobility limitations may help reduce the risk of subsequent loneliness. Physical recovery does not restore social and emotional well-being. For those with persistent mobility limitations, targeted efforts to curb increasing loneliness trajectories could be especially beneficial.

As more people reach old age, increasing numbers of older adults live with mobility limitations that alter their everyday lives. Such limitations reduce the ability to move freely outside the home and maintain social relationships and community independence, increasing the risk of social isolation and loneliness (Pagan & Malo, 2024; Schafer, 2018). Loneliness is a subjective and negative experience arising from a discrepancy between the desired and actual quantity or quality of social relationships (Perlman & Peplau, 1981). It has received increasing attention as a potential modifiable risk factor for several health outcomes (Holt-Lunstad, 2017; Park et al., 2020). While loneliness tends to increase in older age, age itself is not necessarily the primary driver (Dahlberg et al., 2022). Rather, this association appears largely attributable to age-related risk factors, including spousal loss and declining health (Hawkley & Kocherginsky, 2018; Pagan & Malo, 2024). Despite much research on the risk factors of loneliness, longitudinal studies with loneliness as the outcome are lacking (Dahlberg et al., 2022; Meehan et al., 2023; Pagan & Malo, 2024).

## Mobility limitations and loneliness

The onset of mobility limitations often represents a significant transition in later life, as new challenges and increased dependence on others change the conditions of everyday life. As the ability to move freely outside the home declines, older adults may experience reduced contact with friends, family, and their community, which can lead to involuntary social isolation and increased feelings of loneliness (Rantakokko et al., 2013; Tomida et al., 2024).

Severe limitations are consistently associated with higher levels of loneliness, but findings regarding less severe limitations are mixed in longitudinal analyses (Dahlberg et al., 2022). Mobility and loneliness likely influence each other: mobility limitations may increase loneliness, while persistent loneliness may contribute to declines in mobility, creating a cycle (Barjaková et al., 2023; Pollak et al., 2023). However, relatively few studies have examined how mobility limitations, or the onset of limitations, influence loneliness trajectories (Pagan & Malo, 2024; Pollak et al., 2023). Focusing on the onset of mobility limitations allows for a clearer temporal perspective, helping to address bidirectionality and better capture how changes in health may lead to subsequent changes in loneliness. Given the heterogeneity of the aging process and the common occurrence of mobility loss in later life (Ram et al., 2010), a longitudinal perspective is needed.

Mobility decline itself is highly heterogeneous. It may develop gradually over many years, or suddenly due to a critical event, such as a hip fracture following a fall. While some individuals recover, particularly at younger ages, others experience persistent limitations (Köhler & Vanhoutte, 2024; Latham et al., 2015). Recovery is less likely among older, frail, or cognitively impaired older adults (Gill et al., 2006; Manini, 2013), yet approximately one in four older adults regain independence in activities of daily living within 2 years, depending on the severity at onset (Federman et al., 2010). Social support, physical activity, and overall health can buffer or even reverse the progression of mobility decline (Gyasi et al., 2020; Latham et al., 2015).

Whether limitations persist or are temporary is likely important for experiences of loneliness. Short-term limitations may cause temporary disruptions and allow older adults to return to previous levels of social participation, whereas persistent limitations can lead to more permanent reductions in social contact and increased feelings of loneliness. Declines in mobility can reduce both the ability and motivation to maintain social ties, suggesting that persistent mobility limitations may have particularly strong implications for loneliness (Huxhold et al., 2022). Thus, understanding these potentially divergent trajectories is important when examining loneliness trajectories surrounding mobility limitation onset.

## The current study

The overall aim of this study is to examine how loneliness evolves in relation to mobility limitation onset, focusing on two specific research questions: (1) How do loneliness trajectories evolve surrounding the onset of mobility limitations? (2) How do loneliness trajectories differ by the duration of mobility limitations?

## Methods

### Sampling frame and participants

We used data from the Survey of Health, Ageing and Retirement in Europe (SHARE) to examine how loneliness evolves in relation to the onset of mobility limitations. SHARE is a cross-national and longitudinal panel survey that collects data on older Europeans’ social and economic circumstances and health through computer-assisted face-to-face interviews (except in wave 9, where the mode changed to telephone). The data comprises panel data on a large sample of individuals aged 50 and older across 27 European countries and Israel, collected biennially.

One age-eligible respondent is randomly selected per household, along with their partner, regardless of age. To maintain representation over time, refreshment samples are regularly added. Response rates generally vary between 40% and 70% depending on the country and wave (Börsch-Supan et al., 2013). Our analytic sample includes respondents aged 65+ from waves 4 (2011) to 8 (2020).

Information on mobility limitations from wave 9 was retained, allowing us to classify the duration of limitations for individuals who experienced onset in wave 8, without introducing the potential confounding effects of COVID-19 on reported loneliness in wave 9 (collected in 2021-22). For wave 8, we only use data collected before COVID-19.

### Design

Onset of limitations was defined as the first wave in which an individual reported any mobility limitation (T0). People whose first observation was with limitations were excluded from the sample, as the time of their limitation onset could not be identified. Based on mobility status 2 years following onset (T2), respondents were categorized into two groups (totalling 33% of the analytical sample): Remaining (51%) who still reported mobility limitations in T2, and Recovered (49%) who no longer reported limitations in T2.

The reference group (67% of the analytical sample) consisted of participants who did not experience mobility limitations and therefore had no onset; as such, their first observation was treated as their equivalent of pre-onset (T-2).

Time was measured in 2-year intervals relative to mobility limitation onset (T0), with up to four observations per person (T-2–T4). Respondents who switched between “Remaining” and “Recovered” between T2 and T4 were included, but data from T4 were excluded to avoid misclassification. For an overview of the data structure in relation to the SHARE waves, see Table S1.

Anchoring time around the onset allows for the estimation of pre- and post-onset trajectories relative to the event, rather than age, improving causal interpretability of changes surrounding mobility limitation (Köhler & Vanhoutte, 2024). See Figure S1 for the flowchart of the sample selection process.

In wave 7 (in 2017), a substantial number of participants did not receive questions on loneliness (Bergman et al., 2019). To maximize the sample size and minimize selection bias, individuals with missing information on loneliness in the wave following the onset of mobility limitations were included in the analyses, provided that information on mobility limitations was available in T2. Thus, only two waves were considered for these individuals (T-2 and T0), while still allowing classification into remain and recover groups.

The final sample consisted of 4,109 individuals experiencing a mobility limitation onset and 8,197 in the reference group from the following 16 countries: Austria, Belgium, Czech Republic, Denmark, Estonia, France, Germany, Greece, Israel, Italy, Luxembourg, Poland, Slovenia, Spain, Sweden, and Switzerland.

### Variables

#### Loneliness

Loneliness was measured using the three-item University of California, Los Angeles (UCLA) loneliness scale, which is a widely used and validated scale based on information related to intimate, relational, and collective connectivity (Hughes et al., 2004). Loneliness was measured in the mental health section of SHARE for waves 5–8, and in the drop-off questionnaire in wave 4; these have previously been combined to look at trajectories of loneliness (Pagan & Malo, 2024). Questions included in the scale were “How much of the time do you feel you lack companionship?”, “How much of the time do you feel left out?”, and “How much of the time do you feel isolated from others?”. Response options were reverse-coded [Hardly ever or never (0), Some of the time (1), Often (2)] and summarized to create an index (0-6). In line with previous use of this scale, we considered loneliness as a continuous outcome (Marquez et al., 2023).

#### Mobility limitations

Mobility limitations were measured as a binary variable indicating difficulties expected to last over 3 months with any of the following: walking 100 m, walking up a flight of stairs, or rising from a chair (“Please tell me whether you have any difficulty doing each of the everyday activities on card 10. Exclude any difficulties that you expect to last less than three months”; response options: Yes/No). These three items were selected because they affect independence and the ability to participate in social settings (Kim et al., 2012; Moeyersons et al., 2022).

The measure does not capture severity or number of difficulties; any reported limitation meeting the criteria was coded as present.

#### Control variables

Key confounders were added to adjust for differences across groups. These included sex, country, age (65–96), and education level [three categories: lower (less than upper secondary), middle (upper secondary and vocational training), higher (tertiary education)]. Living alone was assessed by household size, where households with one person were coded as living alone. This was treated as a time-varying covariate. These covariates were chosen because they are well-known risk factors for both loneliness and mobility limitations, and country differences in loneliness and mobility levels are known from comparative studies.

Self-rated health was used as a matching variable in sensitivity analyses (“Would you say your health is” with response options: Poor = 0 to Excellent = 4).

### Data analyses

Descriptive statistics were produced to examine group composition. Differences between groups were tested using *t*-tests for continuous variables and chi-square tests for categorical variables.

To examine nonlinear trajectories of loneliness in relation to the onset of mobility limitations, we combined spline and multilevel growth models (Köhler & Vanhoutte, 2024). Because loneliness data could be missing at T2, multilevel growth models were chosen. These models include all available observations for each participant and accommodate unbalanced panels, increasing statistical power and precision in estimating trajectories. The growth models included random intercepts and slopes to capture individual differences in pre-onset (T-2) loneliness and change over time. Time was divided into distinct segments, capturing changes in loneliness as mobility onset occurs (T-2 to T0) and changes following onset (T0 to T4). By using linear splines, we estimated a continuous function that changes slope at specified knots; this allowed for flexible yet interpretable trajectories without assuming constant linear change over the entire period. Positive slopes indicate increases in loneliness, while the coefficient reflects the degree of change.

In the first model, we introduced a level-1 interaction between each segment of the spline model and the indicator of the onset of mobility limitations to examine whether the loneliness trajectory differs between persons who experience the onset of mobility limitations and those who do not. In model 2, we focused on the group experiencing mobility limitation onset and added an interaction between duration of limitations and the splines to investigate how the temporal dynamics of loneliness might vary. Both models were adjusted for living alone as a time-varying covariate and pre-onset (T-2) country, sex, age, and education.

Analyses were performed in Stata (Version 18.0) using maximum likelihood estimation. Ethical approval for the study was granted by the Swedish Ethical Review Authority (2025-03046-01).

## Results

The sample characteristics within each group are presented in Table 1. The groups differ significantly for most selected variables; only the sex distribution does not differ significantly between the recover and remain groups. The proportion of older adults living alone increased over time in all groups. Levels of loneliness differed across groups prior to onset, with the reference group exhibiting the lowest levels and the remaining group the highest. Overall, loneliness levels were relatively low across all groups, with mean values ranging from 0.5 to 1.5 across groups on a 0–6 scale. This indicates that the majority of individuals reported no or only occasional loneliness.

**Table 1:** Descriptive statistics of the sample.

Variables	Total	Onset of mobility limitations			Duration of mobility limitations		
		Reference^a^	Onset	Test	Recover	Remain	Test
**∼2 years pre-onset (T-2)**							
*N*	12,306	8,197	4,109		2,018	2,091	
Loneliness	0.62 (1.11)	0.51 (0.98)	0.85 (1.29)	<.001	0.76 (1.21)	0.93 (1.36)	<.001
Female	51%	46%	59%	<.001	58%	61%	.12
Lives alone	23%	21%	29%	<.001	25%	32%	<.001
Age	71.20 (5.87)	69.88 (5.07)	73.80 (6.45)	<.001	72.77 (5.90)	74.79 (6.79)	<.001
Education: low	39%	34%	50%	<.001	48%	53%	.006
Education: medium	32%	33%	30%		31%	28%	
Education: high	29%	33%	20%		21%	19%	
**At onset (T0)**							
*N*	12,306	8,197	4,109		2,018	2,091	
Loneliness	0.75 (1.22)	0.58 (1.05)	1.08 (1.45)	<.001	0.96 (1.33)	1.21 (1.55)	<.001
Lives alone	25%	22%	31%	<.001	27%	35%	<.001
Limitation: stairs			26%		19%	33%	<.001
Limitation: chair			65%		66%	65%	.49
Limitation: walk			40%		35%	44%	<.001
Number of limitations			1.31 (0.60)		1.20 (0.49)	1.41 (0.67)	<.001
**∼2 years after onset (T2)**							
*N*	6,998	4,730			1,108	1,160	
Loneliness	0.78 (1.23)	0.61 (1.06)		<.001	0.95 (1.30)	1.31 (1.62)	<.001
Lives alone	29%	27%		<.001	30%	40%	<.001
Limitation: stairs						38%	
Limitation: chair						68%	
Limitation: walk						48%	
Number of limitations						1.53 (0.73)	
**∼4 years after onset (T4)**							
*N*	4,616	3,609			495	512	
Loneliness	0.72 (1.21)	0.60 (1.08)		<.001	0.85 (1.24)	1.43 (1.70)	<.001
Lives alone	29%	26%		<.001	33%	44%	<.001
Limitation: stairs						50%	
Limitation: chair						73%	
Limitation: walk						53%	
Number of limitations						1.76 (0.80)	

*Note*. Range: loneliness (0–6); number of limitations (0–3). *P*-values are based on *t*-tests for continuous variables and chi-square tests for categorical variables.

a The reference group refers to participants who did not experience mobility limitations and thereby had no onset.

The reference group (those who never experienced mobility limitations) was generally younger, consisted of more men, had higher levels of educational attainment, and had fewer persons living alone. Differences between the recover and remain groups were generally modest but consistent, with the remain group showing slightly higher levels of loneliness, a higher proportion living alone, and a higher number of mobility limitations across follow-up waves.

**Figure 1:**
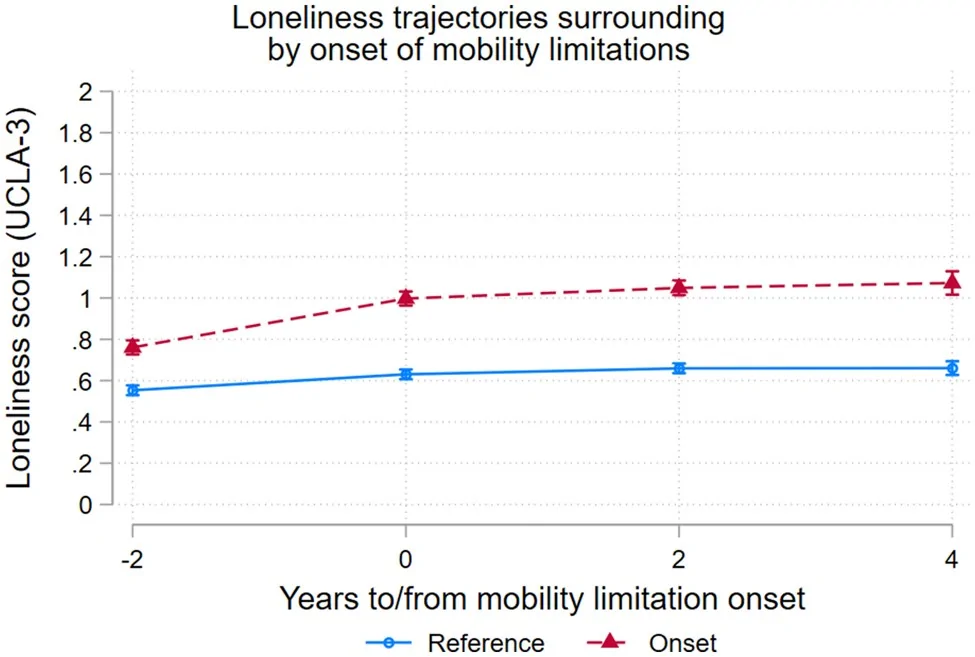
Loneliness trajectories for groups experiencing the onset of mobility limitations, and groups with no mobility limitations. Predicted loneliness trajectories of the reference and onset group; range of loneliness is 0–6.

Figure 1 shows the predicted loneliness for the onset and reference groups from Model 1 in Table 2. The results indicate a statistically significant increase in loneliness from pre-onset to onset, as well as a smaller increase following onset. Individuals experiencing onset had a larger increase in loneliness between T-2 and T0 compared to those not experiencing onset (b = .08, 95% CI [.06, .10]). In contrast, differences in change between groups in the period following onset were small (b = .02, 95% CI [−.01, .03]).

**Table 2:** Growth models assessing change in loneliness (0–6) surrounding mobility limitation onset.

Variables	Model 1	Variables	Model 2
Onset (ref. reference)	0.21	Remain (ref. recover)	0.10
	[.16, .25]		[−.02, .18]
T-2 → T0	0.03	T-2 → T0	0.09
	[.02, .05]		[.06, .12]
T0 → T4	0.01	T0 → T4	−0.01
	[.00, .02]		[−.03, .02]
Onset × T-2 → T0	0.08	Remain × T-2 → T0	0.03
	[.06, .10]		[−.01, .08]
Onset × T0 → T4	0.02	Remain × T0 → T4	0.05
	[−.01, .03]		[.02, .09]
*N* Observations	36,213	*N* Observations	11,493
*N* Individuals	12,301	*N* Individuals	4,109

*Note*. Models are adjusted for sex, age, education, country, and living alone as a time-varying factor. ref. = reference.

**Figure 2:**
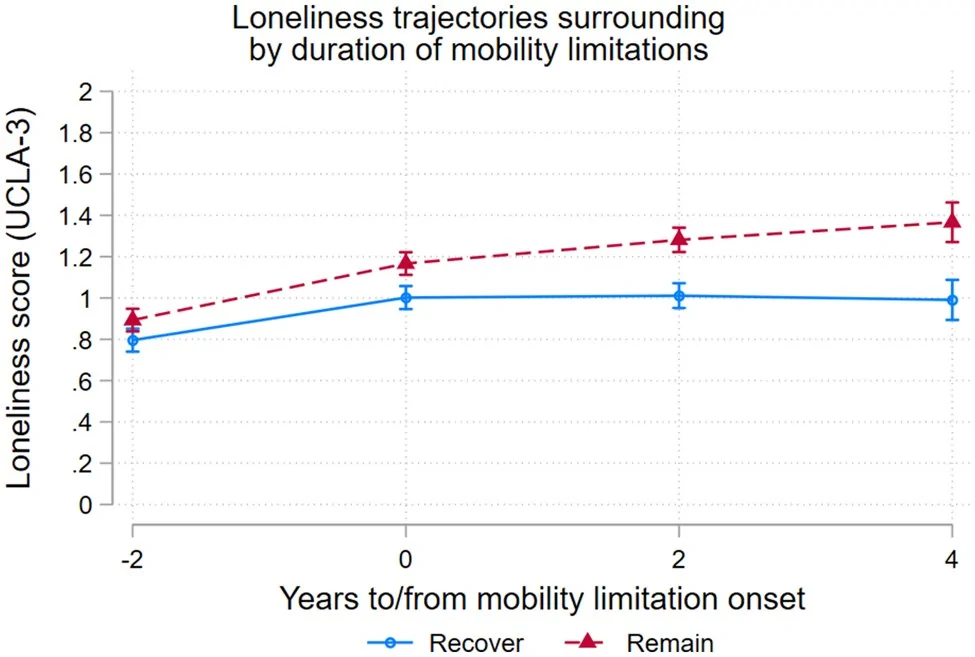
Loneliness trajectories for groups recovering from mobility limitations, and groups with persistent mobility limitations. Predicted loneliness trajectories of the recover and remain groups; range of loneliness is 0–6.

Model 2 differentiates by duration of limitations within the onset group, that is, among those whose mobility limitations remained and those recovering. Loneliness increases between T-2 and T0 across groups (b = .09, 95% CI [.06, .12]), with no statistically significant difference between the recover and remain groups during this period (b = .03, 95% CI [−.01, .08]). In the period following onset, however, trajectories differ: individuals who remain with mobility limitations experience a greater increase in loneliness compared to those who recover (b = .05, 95% CI [.02, .09]). As illustrated in Figure 2, loneliness stabilizes in the recovery group, whereas it continues to increase among those who remain with limitations.

## Sensitivity analysis

To assess potential selection bias arising from the study design, we distinguished between design-related exclusion and attrition after onset (T0) among individuals with observed onset of mobility limitations. Because classification into recover and remain groups requires observations at T2, individuals who dropped out after onset cannot be classified and are therefore structurally excluded from the analytical sample.

Table S2 shows that most exclusions were due to study design (e.g., end of study period, SHARELIFE participation), with a smaller proportion due to attrition.

Comparisons between included individuals and those lost to follow-up at T2 show some differences: attrition cases had higher baseline loneliness, were slightly older, more often male, had lower levels of education, and reported poorer self-rated health and more mobility limitations at T0 (Table S3). Multivariate analyses indicated that these characteristics were modestly associated with attrition (Table S4). While these associations are small in absolute terms, they indicate some degree of selective loss to follow-up among individuals in poorer health. As those who experienced onset but dropped out before this could be captured are not part of the sample, this suggests that the estimated trajectories may represent conservative estimates of changes in loneliness following onset.

Propensity score matching was used as a robustness check to ensure that observed differences in loneliness trajectories are driven by mobility limitations, rather than confounding factors. Matching on baseline characteristics, including loneliness and self-rated health, substantially improved covariate balance, particularly when applying a caliper of .25 (Table S5). Results from the matched samples were highly similar to those from the main analyses (Table S6), indicating that findings are not driven by baseline differences between groups.

Analyses were also re-run on the sample stratified by type of mobility limitation. The separate analyses by mobility type showed similar results, though with smaller samples (Table S7). Contrast analyses by country showed only minor differences (Table S8), suggesting the pooled estimates are robust.

## Discussion

This study set out to examine trajectories of loneliness surrounding the onset of mobility limitations among older adults. Overall, loneliness increased among people who experienced mobility limitations compared to the reference group with no onset. When distinguishing between individuals who recovered from these limitations and those whose limitations persisted, the remaining group’s loneliness continued to increase, while the recovery group’s levels stabilized.

Our first research question asked how the onset of mobility limitations is associated with loneliness trajectories. Comparing all who experienced onset with the reference group, we observed an increase in loneliness surrounding onset. The onset of mobility limitations can mark a significant change, impacting many aspects of older adults’ lives, including social relationships and feelings of loneliness. While loneliness increased immediately following the onset, the reference group also showed gradual increases over time, indicating an aging-related pattern (Dahlberg et al., 2022).

Although the levels of loneliness remain relatively low following onset (around 1.1 on a 0-6 scale), there is a statistically significant change in loneliness from T-2 to T0. This is likely mostly a transition at the lower end of the range, for example, individuals moving from reporting no to occasional feelings of loneliness. This suggests that the onset of mobility limitation may mark the beginning of increased vulnerability to loneliness, even if individuals do not reach high levels on the scale. While there is no consensual cutoff for loneliness for the UCLA Loneliness Scale, the low mean levels observed across groups are in line with previous research (Surkalim et al., 2022).

We extended the analysis by distinguishing between individuals who recovered from mobility limitations and those whose limitations persisted, as previous research has shown different trajectories of well-being (Köhler & Vanhoutte, 2024) and loneliness (Pagan & Malo, 2024) depending on whether the limitations were transient or not.

On average, loneliness trajectories differed between these groups. While onset was associated with increased loneliness in both groups, increases continued only in the remaining group. Individuals who recovered did not experience a decline in loneliness; instead, levels stayed elevated relative to pre-onset but stabilized thereafter, suggesting that recovery in physical function does not necessarily translate into improvements in social or emotional well-being.

This pattern is consistent with previous research linking health problems to loneliness (Dahlberg et al., 2022), but contrasts with patterns of well-being following the onset of disability (Köhler & Vanhoutte, 2024), where recovery is often accompanied by a return to the previous level of well-being.

Mobility limitations can alter daily life and the ability to maintain social connections. Social participation may require greater effort as individuals accommodate new limitations (Huxhold et al., 2022). Barriers such as transportation, accessibility, and self-consciousness about imposing on others may increase. Evidence suggests that people with disabilities sometimes withdraw from interactions or perceive themselves as burdensome, which may reinforce loneliness (Moeyersons et al., 2022; Pedroso-Chaparro et al., 2023). However, social networks can also be mobilized in response to health decline, sometimes referred to as social network activation (Latham-Mintus, 2019; Perry & Pescosolido, 2015). In these cases, discussion networks may expand, or individuals may become more active in their relationships, though this can be highly context-dependent (Schafer, 2018). Moreover, trajectories of psychosocial resilience following functional loss are associated with higher levels of social connection (Agahi et al., 2024). Together, these findings suggest that maintaining social connectedness during periods of health decline may be important for preserving mental well-being and preventing sustained increases in loneliness.

### Strengths and limitations

The results of this study should be interpreted in light of some strengths and limitations. Regarding the outcome measure, loneliness is captured through a well-established measure, which has been shown to perform as well as the 20-item-scale to measure the distribution of feelings of loneliness in the older population (Hughes et al., 2004). However, a substantial proportion of our sample had missing information on loneliness data, primarily because they did not receive the question in wave 7 of SHARE. This study design created a loss of information at T2 (4,303 individuals) and T4 (1,842 individuals), resulting in an overrepresentation of persons with only two measurement points.

As our study is observational, it limits our ability to draw causal conclusions, and the possibility of unmeasured confounding cannot be ruled out. Even so, the clearly defined timing of mobility limitation onset and the inclusion of a comparison group provide a stronger basis for cautious causal interpretation. Baseline differences may have influenced the observed trajectories: individuals who experienced onset had higher loneliness even before onset, likely reflecting shared correlates of loneliness and mobility limitations. Although the models adjusted for several observed covariates, residual confounding may remain. However, propensity score matched analyses, incorporating baseline loneliness and self-rated health, improved comparability between groups and yielded substantively similar results to the main models, suggesting that the findings are not primarily driven by observed baseline compositional differences.

While the onset of mobility limitation was anchored between two time points, individuals may have experienced it at any point within that interval. Some participants likely had nearly 2 years to adapt to their new circumstances, while others may have had only weeks, potentially attenuating observed associations or obscuring short- vs long-term effects. In addition, classification into the remain and recover groups required observation at T2, meaning that individuals lost to follow-up after onset were structurally excluded from the analytical sample. Individuals most severely affected by mobility decline may therefore be underrepresented, introducing potential selection bias, which could contribute to an underestimation of loneliness levels following onset. Supplementary analyses further indicated some degree of selective attrition, as individuals lost to follow-up tended to report poorer health and higher loneliness at baseline. Although these differences were modest in magnitude, and matched analyses yielded substantively similar findings, some selection bias due to unobserved factors may remain.

Data were collected across 16 countries, which likely differ in the prevalence of loneliness, the consequences of mobility limitations, and the social network structures. Sensitivity analyses suggested that findings were not driven by any single national context. The findings should therefore be interpreted as reflecting average patterns across a diverse European population.

## Conclusion

Loneliness trajectories following the onset of mobility limitations highlight that both functional decline and its social consequences are dynamic. Individuals with persistent limitations are at particular risk of loneliness, and even those who recover physically may remain socially or emotionally vulnerable. Mixed findings regarding the social and emotional well-being trajectories of individuals who recover from functional decline require further investigation. Recognizing the transient nature of mobility limitations, early interventions that support social participation may help prevent worsening loneliness, particularly for those with persistent limitations.

## Supplementary Material

gbag155_Supplementary_Data

## Data Availability

SHARE data are readily available through the SHARE homepage (http://www.share-project.org). The study was not preregistered.
