# Supplementary material for: Loneliness trajectories surrounding mobility loss: the role of disability duration
Source: J Gerontol B Psychol Sci Soc Sci. 2026 Aug 5;81(10):gbag155. doi: 10.1093/geronb/gbag155 (PMC13597101; doi:10.1093/geronb/gbag155)
Supplement: Supplementary file 1 [file gbag155_supplementary_data.pdf]

The Journals of Gerontology, Series B: Psychological Sciences and Social Sciences
Supplementary Material: Augustsson et al. Loneliness trajectories surrounding mobility loss: The role of disability duration.

Contents

Figure S1. Flow chart of sample selection 2

Table S1. Distribution of Observation Window Patterns by Group 2

Selection analyses 3

Table S2. Reason for exclusion among those excluded 3

Table S3. Attrition (loss after T0) Characteristics at T0 3

Table S4. Average Marginal Effects of belonging in the attrition group. 4

Matching analyses 4

Table S5. Covariate balance before and after matching for the onset and duration samples. 5

Matching results 5

Table S6. Models assessing change in loneliness (UCLA 0-6) surrounding mobility limitation onset, coefficient, and 95%CI. 6

Additional robustness checks 7

Table S7. Analyses by mobility type. 7

Table S8. Contrast analysis on the onset group using the observation-weighted grand mean. 8

Figure S1. Flow chart of sample selection

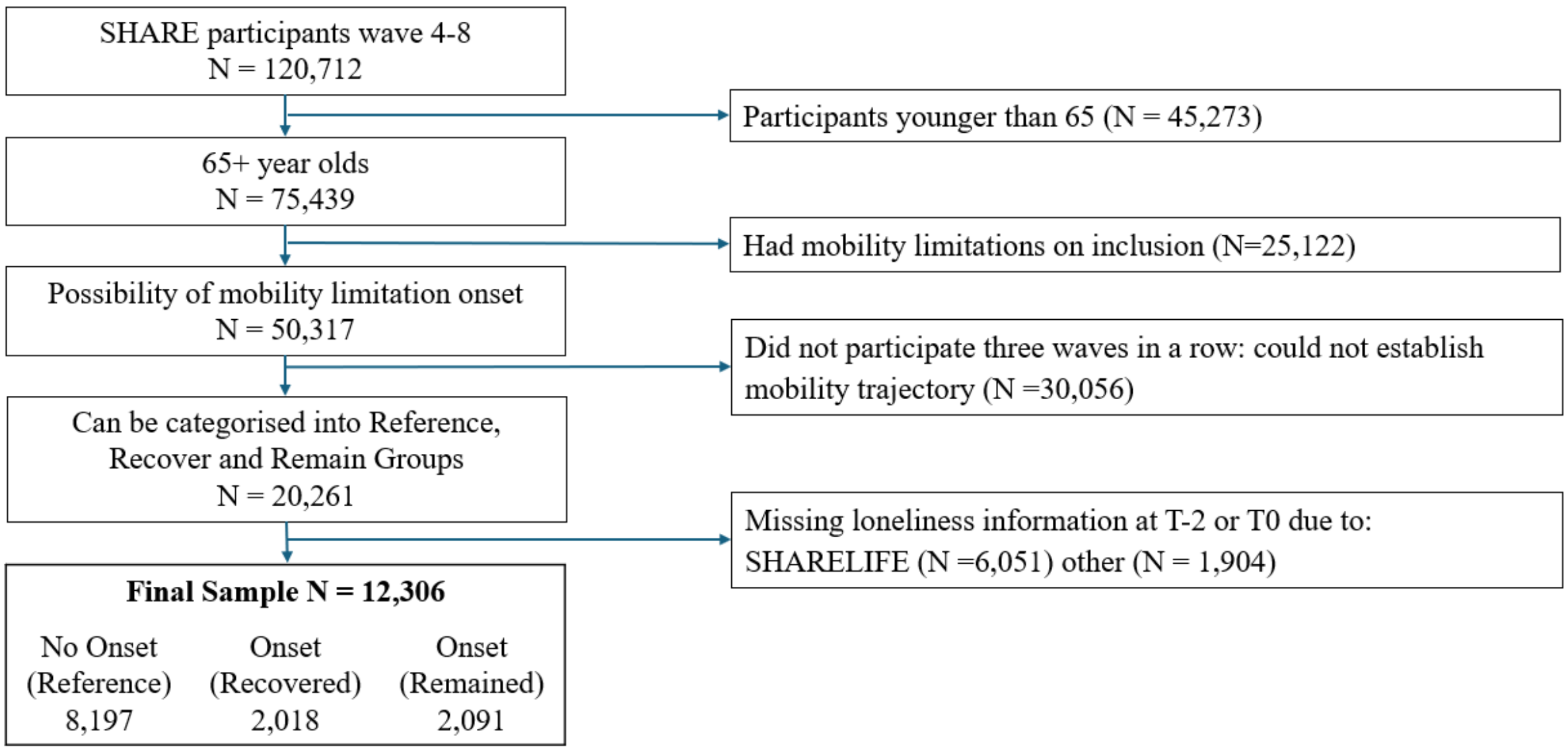

Table S1. Distribution of Observation Window Patterns by Group

Observation window pattern (waves relative to onset)	Experienced onset (%)	Reference (%)
W4 (−2), W5 (0)	0.5	1
W4 (−2), W5 (0), W6 (2)	24	24
W4 (−2), W5 (0), W6 (2), W7 (4)	19	8
W5 (−2), W6 (0)	15	22
W5 (−2), W6 (0), W7 (2)	3	8
W5 (−2), W6 (0), W7 (2), W8 (4)	4	5
W5 (−2), W6 (0), W8 (4)	21	12
W6 (−2), W7 (0), W8 (2)	8	11
W7 (−2), W8 (0)	6	9

Selection analyses

To assess potential selection bias, we compared individuals included in the analytical sample to those who experienced limitations but were excluded due to missing information at T2, restricting all analyses to individuals with observed onset.

To distinguish between design-related exclusion and attrition, excluded individuals were classified according to their reason for exclusion (Table S2).

Table S2. Reason for exclusion among those excluded

Reasons	*N*
End of study	2,125
SHARELIFE at T-2/T0	3,820
Missing loneliness	1,225
Attrition	568

Among individuals eligible for follow-up, those lost to follow-up at T2 showed modest differences compared to those retained in the analytical sample (Table S3). Attrition cases were slightly older, more often male, reported poorer self-rated health, lower educational attainment, higher baseline loneliness, and a greater number of mobility limitations at T0.

Table S3. Attrition (loss after T0) Characteristics at T0

	Included	Attrition	Test
N	4,109	571	
UCLA	1.1 (1.4)	1.4 (1.7)	<.001
Age	76 (7)	78 (7)	<.001
Female	59	47	<.001
Living alone	31	31	0.75
Self-rated health	1.4 (0.9)	1.1 (0.9)	<.001
Education: Low	50	57	0.013
Education: Medium	30	26	
Education: High	20	18	
Number of limitations	1.3 (0.6)	1.5 (0.7)	<.001

To assess the magnitude of these differences, a mutually adjusted logistic regression model predicting attrition was estimated (Table S4). Average marginal effects indicated that associations were generally small. The strongest association was observed for sex, with men being more likely to drop out. Health-related variables (self-rated health and number of limitations) were also associated with attrition, although effect sizes remained modest.

Table S4. Average Marginal Effects of belonging in the attrition group.

Variables	AME	CI
UCLA	.008	[.002; .013]
Age	.005	[.004; .007]
Female	-.047	[-.067; -.027]
Living alone	-.017	[-.038; .004]
Self-rated health	-.024	[-.036; -.013]
Education: Low	REF	
Education: Medium	-.012	[-.034; .010]
Education: High	-.015	[-.040; .009]
Number of limitations	.032	[.018; .045]

Overall, these results indicate that attrition is not entirely random, but differences are small in magnitude and unlikely to meaningfully bias estimated trajectories. Estimates should nevertheless be interpreted as potentially conservative with respect to loneliness following onset and likely underrepresenting persons in poor health.

Matching analyses

Propensity score matching was used as a robustness strategy to assess whether differences in loneliness trajectories were driven by baseline compositional differences between groups. Matching was done separately for the onset and duration samples at T0 using age, sex, country, education, baseline self-rated health, baseline living situation, and baseline loneliness.

Matching was implemented in Stata using psmatch2 and nearest-neighbour 1:1 matching without replacement. This specification retains all individuals in the onset group but excludes unmatched individuals from the reference group.

As a robustness check, we additionally used the same matching procedure and specified a caliper (0.25 standard deviations of the propensity score) to ensure matches were drawn from regions of common support and to improve covariate balance. This improved covariate balance but reduced sample size (3,215 matched pairs in the onset analysis, 894 unmatched in the onset group; 1,812 matched individuals in the duration analysis, 279 unmatched in the remain group).

Covariate balance was assessed using standardized mean differences (SMD) as well as Rubin's B and R statistics. Following caliper matching, all covariates achieved satisfactory balance, with SMDs below 0.07 (Austin, 2009), Rubin's B below 25 and Rubin's R between .5and 2) statistics (Table S5) (Rubin, 2001). In contrast, matching without a caliper showed residual imbalance for several covariates.

Table S5. Covariate balance before and after matching for the onset and duration samples.

Variables	Onset			Duration		
	Unmatched SMD	1:1 SMD	Caliper (.25) SMD	Unmatched SMD	1:1 SMD	Caliper (.25) SMD
UCLA	0.29	0.15	0.00	0.13	0.11	0.01
Age	0.68	0.34	-0.03	0.32	0.25	0.02
Women	0.26	0.09	0.01	0.05	0.04	0.00
Self-rated health	-0.76	-0.19	0.04	-0.25	-0.21	-0.04
Live alone	0.19	0.10	0.00	0.17	0.13	0.01
Education: Medium	-0.07	-0.06	0.01	-0.05	-0.05	-0.02
Education: High	-0.30	-0.06	-0.01	-0.07	-0.06	-0.01
Germany	-0.05	-0.03	0.01	0.02	0.02	0.00
Sweden	-0.21	-0.05	-0.01	-0.09	-0.08	0.00
Spain	0.10	0.02	0.00	-0.04	-0.04	-0.03
Italy	0.09	0.01	0.02	0.00	0.00	0.00
France	-0.01	0.00	0.01	0.04	0.03	0.01
Denmark	-0.15	-0.02	-0.01	0.04	0.02	0.01
Greece	0.09	0.04	0.01	0.05	0.05	0.01
Switzerland	-0.10	-0.02	0.02	-0.02	-0.02	0.00
Belgium	0.02	-0.01	-0.01	0.00	0.00	0.00
Israel	-0.13	-0.02	0.02	-0.03	-0.03	-0.01
Czech Republic	0.07	-0.02	0.00	0.03	0.03	0.01
Poland	-0.03	-0.03	-0.02	-0.03	-0.03	0.00
Luxembourg	-0.02	-0.01	0.00	0.02	0.02	0.00
Slovenia	0.09	0.02	-0.01	0.02	0.02	-0.01
Estonia	0.21	0.14	-0.02	-0.05	-0.04	0.01
Mean Bias	0.18	0.06	0.01	0.07	0.06	0.07
Med Bias	0.10	0.03	0.01	0.04	0.03	0.41
	Rubin's diagnostics			Rubin's diagnostics		
Rubin's B		46.50	8.00		39.4	6.7
Rubin's R		1.91	0.96		0.92	0.88

Matching results

Table S6 presents results from the main models alongside estimates from the matched samples.

For the onset analysis, results from the no-caliper 1:1 matched sample closely resemble those from the main models. The caliper-matched specification yields similar trajectory patterns, with improved baseline balance and slightly attenuated group differences in levels. The main pattern of increased loneliness around onset remains robust across specifications.

For the duration analysis, results are broadly consistent across all models. Differences between the remain and recover groups are reduced in the matched samples, particularly in the caliper specification, but the overall trajectory pattern remains unchanged.

Overall, matching does not materially alter the substantive conclusions but improves baseline comparability between groups.

Table S6. Models assessing change in loneliness (UCLA 0-6) surrounding mobility limitation onset, coefficient, and 95%CI.

	Reference vs. Onset Group				Remain vs. Recover Group		
Duration	Model 1	Fully matched	Caliper	Duration	Model 2	Fully matched	Caliper
Onset	.21	.13	-.01	Remain	.10	.08	.00
	[.16,.25]	[.08,.18]	[-.06,.05]		[.02,.17]	[-.00,.16]	[-.08,.08]
T-2 → T0	.03	.03	.02	T-2 → T0	.09	.09	.09
	[.02,.05]	[.01,.05]	[-.00,.04]		[.06,.12]	[.06,.12]	[.05,.12]
T0 → T4	.02	.02	.02	T0 → T4	.00	.01	.01
	[.01,.02]	[.00,.03]	[.01,.04]		[-.02,.03]	[-.02,.03]	[-.02,.03]
Onset T-2→T0	.08	.08	.10	Remain T-2→T0	.04	.04	.05
	[.06,.10]	[.06,.11]	[.07,.13]		[-.01,.08]	[-.01,.08]	[.00,.10]
Onset T0→T4	.01	.01	.01	Remain T0→T4	.05	.05	.04
	[-.01,.03]	[-.01,.03]	[-.01,.03]		[.02,.08]	[.02,.09]	[.01,.08]
N	12,301	8,218	6,430	*N*	4,109	4,036	3,624

Note. Fully matched sample (all in the exposed group were matched), whereas caliper matched sample only those in the exposed group with a close match in the reference/remain group were matched.

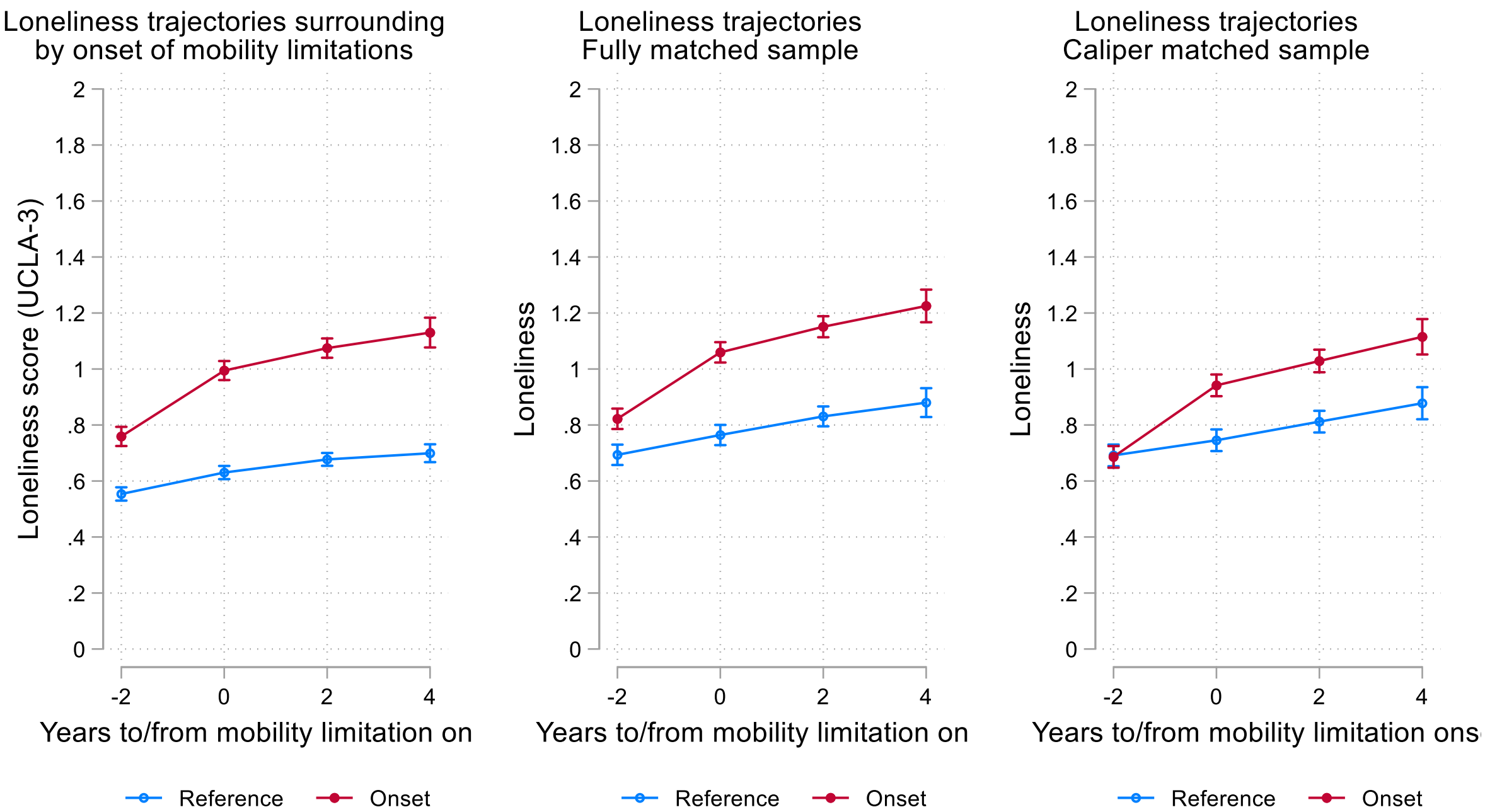

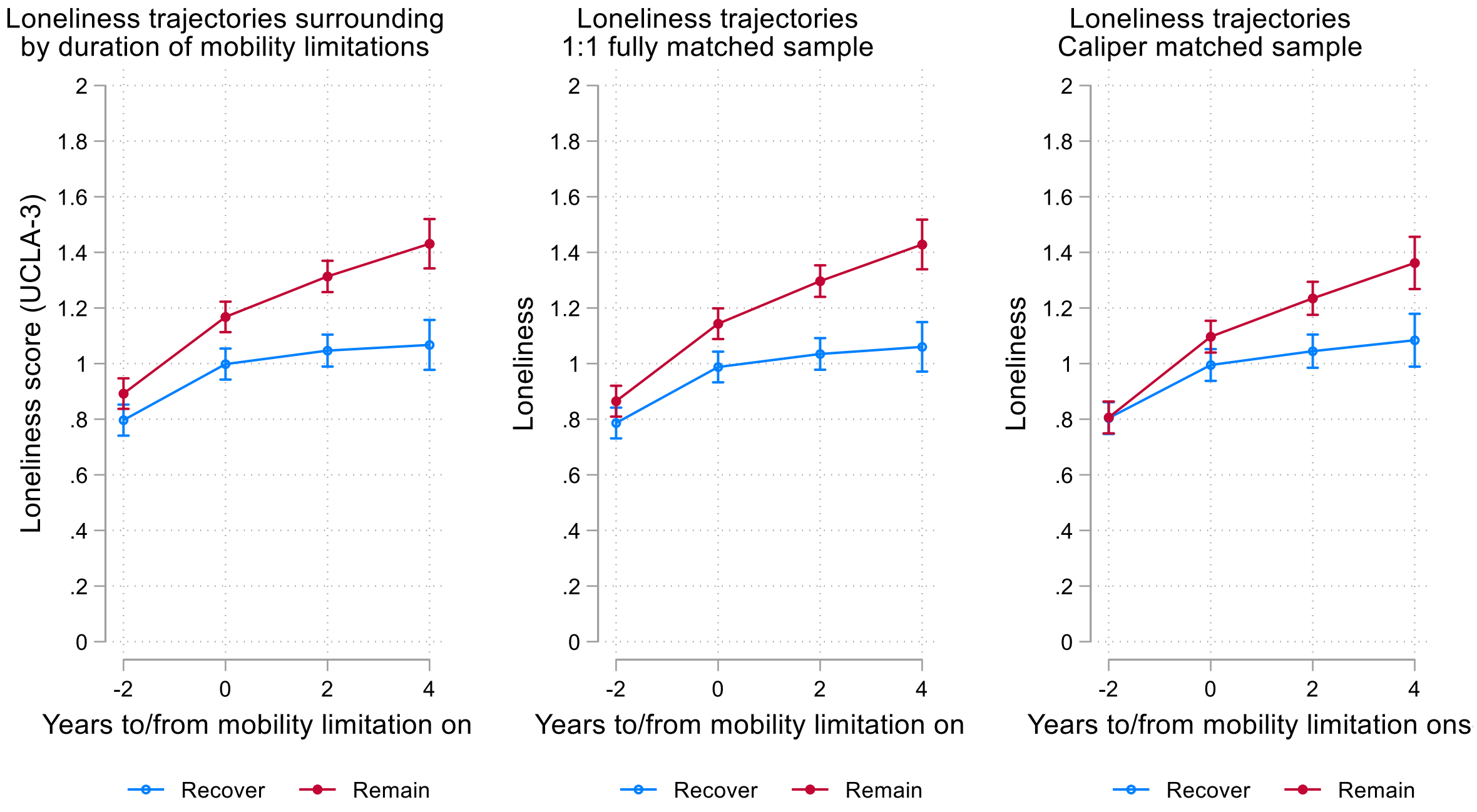

Additional robustness checks

Analyses were additionally rerun separating types of mobility limitations. Results were similar, although estimates were less precise due to smaller sample sizes (Table S7).

Table S7. Analyses by mobility type.

Duration	Stairs	Chair	Walk	Duration	Stairs	Chair	Walk
Onset	.17	.15	.16	Remain	0.05	.09	0.14
	[.12,.23]	[.11,.19]	[.10,.22]		[-.08,.18]	[.00,.18]	[-.024,.31]
T-2→T0	.045	.041	.046	T-2→T0	.09	.10	.17
	[.03,.06]	[.03,.06]	[.03,.06]		[.02,.15]	[.06,.15]	[.08,.27]
T0→T4	.015	.017	.017	T0→T4	0.01	-0.01	-0.02
	[.01,.02]	[.01,.03]	[.01,.03]		[-.05,.07]	[-.04,.03]	[-.10,.06]
Onset T-2→T0	.10	.08	.14	Remain T-2→T0	.10	0.03	0.03
	[.06,.14]	[.05,.11]	[.1,.19]		[.01,.19]	[-.03,.09]	[-.09,.15]
Onset T0→T4	0.02	0.00	-0.00	Remain T0→T4	0.04	0.05	0.04
	[-.01,.05]	[-.02,.02]	[-.04,.04]		[-.03,.11]	[-.00,.10]	[-.06,.14]
N	12,301	12,301	12,301	*N*	1,626	2,691	1,067

Country contrast analyses using interaction terms between country and time splines showed generally similar trajectories across countries, with only small deviations in a limited number of cases (Table S8). These differences are unlikely to affect overall conclusions.

Table S8. Contrast analysis on the onset group using the observation-weighted grand mean.

	Contrast	95% CI	
**T-2 à T0 by country**			
Austria	0.01	-0.07	0.08
Germany	-0.03	-0.11	0.05
Sweden	-0.07	-0.16	0.01
Spain	0.04	-0.02	0.11
Italy	**0.16**	**0.09**	**0.23**
France	-0.01	-0.09	0.07
Denmark	**-0.11**	**-0.20**	**-0.02**
Greece	-0.03	-0.14	0.08
Switzerland	-0.01	-0.10	0.07
Belgium	-0.07	-0.15	0.00
Israel	0.17	-0.01	0.35
Czech Republic	0.04	-0.02	0.11
Poland	-0.04	-0.21	0.14
Luxembourg	-0.18	-0.39	0.03
Slovenia	-0.03	-0.12	0.06
Estonia	-0.04	-0.13	0.06
**T0 à T4 by country**			
Austria	-0.02	-0.08	0.05
Germany	-0.02	-0.09	0.04
Sweden	0.04	-0.03	0.10
Spain	0.05	0.00	0.11
Italy	-0.01	-0.07	0.04
France	-0.03	-0.10	0.03
Denmark	0.04	-0.02	0.11
Greece	**-0.24**	**-0.37**	**-0.10**
Switzerland	0.01	-0.06	0.07
Belgium	0.00	-0.06	0.06
Israel	0.04	-0.13	0.22
Czech Republic	0.00	-0.05	0.06
Poland	-0.03	-0.24	0.18
Luxembourg	-0.17	-0.34	0.00
Slovenia	0.04	-0.04	0.12
Estonia	0.06	-0.01	0.13
